# Communications

**Published:** 1873-01

**Authors:** 


*Communications.*

The following communications have been received: "A Case of Incapsulated Pleuritic Effusion," by John E. Lockbridge, M.D.; "Electro-Magnetism in Acute Diseases," by A. Donald, M.D.; "Mykomyringitis, or Myringitis Parasitica," by A. W. Calhoun, M.D.; Correspondence—Baltimore—by Charles S. Lewis, M.D.

Our friends are requested to forward articles. Our circulation guarantees a large number of professional readers in every Southern and many of the Northern States. Send your communications along.

